# Precision pharmacology: deep learning infused ontological framework with E-GRU enhancement for tailored medicine prescriptions

**DOI:** 10.1038/s41598-026-48044-x

**Published:** 2026-08-03

**Authors:** Harichandra Khalingarajah, Asokan Vasudevan, P. Abinaya, T. Manonmani, N. Raja

**Affiliations:** 1 Faculty of Health and Life Sciences, INTI International University, Persiaran Perdana BBN, Putra Nilai, 71800 Nilai, Negeri Sembilan Malaysia; 2 Department of Fisheries, Faculty of Science, University of Jaffna, Ramanathan Road, Thirunelvely, PO. Box 57, 40000 Jaffna, Sri Lanka; 3 International Relations, INTI International University, Persiaran Perdana BBN, Putra Nilai, Nilai, 71800 Negeri Sembilan Malaysia; 4 Faculty of Business and Communications, INTI International University, Persiaran Perdana BBN Putra Nilai, 71800 Nilai, Negeri Sembilan Malaysia; 5 Department of Computer Science and Engineering, Mepco Schlenk Engineering College, Sivakasi, Tamil Nadu India; 6 Department of Computer Science and Engineering, Mepco Schlenk Engineering College, Sivakasi, Tamil Nadu India; 7 Department of Visual Communication, Sathyabama Institute of Science and Technology, Chennai, Tamil Nadu India

**Keywords:** EHR - Electronic health records, ICD - International code of diseases, ATC - Anatomical therapeutic chemical, E-GRU - Enhanced gated recurrent unit, NDCG - Normalized discount cumulative gain, Cancer, Physiology, Diseases, Health care, Health occupations, Medical research, Molecular medicine, Oncology, Signs and symptoms, Mathematics and computing

## Abstract

Advanced Clinical Decision Support Systems significantly influence patient care, with medicine prescriptions being a vital area of research. Ontology, a growing discipline in the semantic web, enables hierarchical domain representation, thereby allowing finer data access to be achieved. Deep Learning (DL) supports pattern recognition in Electronic Health Records (EHR), which include patient demographics and diagnosis histories. Prescribing medications with minimal adverse effects is crucial, particularly for patients who require multiple drugs, as drug interactions can result in more complex conditions. This study introduces an integrated approach that combines Ontology with DL neural networks to improve prescription accuracy. This study proposes NexusOpti, a model featuring an Enhanced Gated Recurrent Unit (E-GRU) layer. To understand drug–disease interactions, hierarchical data were extracted from the International Classification of Diseases (ICD) and Anatomical Therapeutic Chemical (ATC) ontologies. These structured data were processed using a self-attention mechanism to enhance the recommendation precision. This integration not only addresses data security concerns but also improves the accuracy of the medicine recommendations. The model was evaluated using key metrics such as the hit ratio and normalised discounted cumulative gain (NDCG). The NexusOpti model, incorporating the Enhanced Gated Recurrent Unit (E-GRU) layer, outperforms the existing GRU model in terms of NDCG and Hit Ratio metrics. 13% of improvement in performance was oberved to the comparison between NexusOpti with the E-GRU and the GRAM baseline model. These findings highlight the effectiveness of the model in advancing personalised, safer, and data-driven medication prescriptions.

## Introduction

Pharmaceutical recommendation systems are the subject of ongoing research to help doctors make better decisions. The prediction of drugs, as detailed by Tsang et al.^1^, to improve therapeutic medicine prescription is a crucial component of systems intended to deliver medical judgments and assist doctors in prescribing drugs for the future based on intricate medical scenarios. A subfield of health informatics, predictive therapy, offers early warnings for medical risk management, prescription management insights, and treatment planning guidance, all of which can enhance clinical decision-making. Prescribing medication is a complicated procedure which is affected by several variables, including demographic data, medical history, genetic predispositions, and environmental influences^2^. Individuals with more complicated conditions require sophisticated pharmaceutical regimens with multiple drug interactions. Such polypharmacy increases the risk of adverse drug events (ADEs), including drug–drug interactions, allergic reactions, toxicity, organ damage, and reduced therapeutic efficacy, which can result in severe clinical complications or even death^3^. Some of these interactions may not be appropriate and may have detrimental consequences. This study suggests the use of pharmaceuticals which trigger any problems as an outcome. Traditional methods for providing pharmaceuticals often rely on clinical criteria and expert opinions, which may not account for subtle interactions among these components. The dosage of medication without undesirable reactions was aided by considering such intricate connections.

The DL methods by Sarker^4^ solve these problems by having the capacity to analyse large medical datasets to identify minute relationships and treatment results. DL algorithms train neural networks based on a range of patient data and clinical circumstances, enabling them to deliver optimal medication selection based on the distinct characteristics of each patient. Ontology was used to extract the hierarchical dependencies of both drug codes and diagnosed disease codes from ATC^5^ and ICD^6^. The extraction of hierarchical data enriches the granularity of the information, facilitating a deeper understanding of the dataset and enabling more precise medicine. Weiet al.^7^ demonstrated that ICD-9 codes are a reliable way to capture the reality of chronic illnesses using administrative data, and by validating these codes for multimorbidity analysis, they provided the groundwork for using hierarchical disease structures to better understand how different conditions interact and how treatment pathways are formed in the future. However, because of the high variation across people, including differences in diagnostic history, demographics, and drug side effects, establishing treatment pathways is difficult in this disease. This study introduced a novel prescription recommendation framework that incorporates ontology and DL to predict the next drug that can be prescribed in disease treatment pathways. In this study, the baseline models for comparison are GRAM, HAP, and Ontopath. These models were chosen for their relevance in personalized prescription recommendation systems, which aim to address similar challenges in clinical decision support and medication optimization. A higher degree of granularity with this combined approach improves the efficacy of the medical recommendations.

Recurrent neural networks (RNN)^8^, Long Short-Term Memory (LSTM)^9^, and gated recurrent units (GRU)^10^ are widely used DL models for prediction using time-series data. However, these models face challenges that hinder the development of personalised medical recommendations. The Ontopath model proposed by Yao et al.^11^ provides a framework for describing personalised recommendations. This model offers an effective method for integrating several factors into the medication recommendation process. It was built using an embedding, transformer, RNN, and predictor layer. A novel paradigm for prescription medicine that uses a DL model is called NexusOpti. The model imposed in this framework comprises an embedding layer, transformer layer with an encoder and decoder, Enhanced GRU (E-GRU) layer, and a prediction layer. In addition, the E-GRU improved the overall performance of the model compared to the baseline models, including GRAM, HAP, Ontopath, and the existing GRU model. The system was assessed using performance metrics such as NDCG and Hit Ratio. The findings show that the suggested paradigm (NexusOpti with E-GRU) is more effective for prescribing medications than above mentioned baseline models. The developed E-GRU exhibited high learning efficiency and an overall increase in the model performance. The main contributions of this study is represent ICD and ATC codes hierarchically through SPARQL data extraction from ICD and ATC ontologies, respectively. And develop the NexusOpti model using E-GRU. The recommendation score for each drug was calculated, and drugs with high recommendation scores were prescribed.

“Introduction” Section provides a general overview of the problem addressed in the proposed study. This section also describes the major objectives and outcomes of the evolved work, relates to the technologies used in the refinement, and discusses the major outlooks of this study. “Related works” Section provides a brief overview of the existing model developed and its comparison with the proposed work and adds valid information to make the work competent. “Methodology” Section explains the methodology and the technical flow involved in the proposal, which details the step-by-step process, including the application of the equations in “Experimentation” Section, and provides a detailed experimentation of the proposed model, including the number of training and testing data used in constructing the model and the system configuration. “Results” Section presents the results and outcomes of the developed model, actuating the evaluation metrics that provide the model performance. “Conclusion and future work” Section provides a detailed analysis of the security of integrating ontologies and deep learning. The conclusion in “Limitations” Section explains the outlook and enhancements that can be applied to the model to make recommendations with greater depth of knowledge. The references used to construct this model are listed in the references section.

## Related works

Ontology has been a major research area owing to its ability to add semantics to the content. Integrating ontology with DL will make it more effective studied by Li et al.^3^. Tsang et al.^1^ employed entropy regularisation with ensemble deep neural networks, a machine learning approach, to pinpoint certain features that are significant within a broad feature domain space. The flow involved four stages: feature importance grouping and ranking, snapshot ensemble training and aggregation, entropy weight regularisation, and backward stepwise feature selection and validation. Codes from patient records over a given timeline were used in the data preprocessing step. Each distinct read code was one-hot encoded as a separate feature. Shoaip et al.^12^ developed an ontology-based fuzzy Clinical Decision Support System (CDSS) for making decisions while diagnosing Alzheimer’s disease (AD), which includes the construction mechanism and ontology evaluation of the crisp AD ontology (ADDO). The methodology uses a knowledge base that consists of a fuzzy ADDO and a rule-based model using SWRL, fuzzification, fuzzy inference, defuzzification, a query engine, and a reasoning engine. The developed ontology was compared with existing AD ontologies. Zhou et al.^13^ implemented a novel ontology model called RTO for multi-domain knowledge organisation. This paper presents a novel ontology model called RTO for multi-domain knowledge organisation. A method for building an RTO based on an adaptive filter was presented and verified. The evaluation criteria for RTO were accuracy, recall, correctness, and runtime. The experimental results show that RTO has higher correctness and lower time consumption than other models.

The implementation of Saeed et al.^14^ explains the method proposed for recognising and assessing major symptoms and infectious diseases. A computational model was used to estimate the severity of infectious diseases. Clinical Nursing Health System (CNHS) mapping was used to identify relevant medications based on severity. A generalised mapping system was used to determine the patient’s status and anticipate prescription needs. This is an effective and beneficial method for identifying infections, but it also faces certain drawbacks, including mapping requirements that should be self-contained within the same structural class; otherwise, they must be converted into base parameters. The methodology detailed by Song et al.^15^ proposed a neural network with external memories to improve the medication prediction performance. The Model can be modified to learn the phases of the disease in an unsupervised manner. Different phases indicate the progression of medications and have distinct signs and symptoms. Neural networks with memory have the potential to learn temporal data. This Model can also be applied to other domains that require information storage. Smirnov et al.^16^ proposed a methodology that involves the integration of multiple domain ontologies for interoperability between humans and machines. Applicability of the methodology in the “e-tourism” domain. This Facilitates ontologies for complex knowledge-based systems operating in multiple domains; however, this methodology lacks the interoperability of existing decision support systems. Existing ontology development methodologies fail to support cross-domain knowledge integration. This also leads to restrictions on the integration approach owing to the structure of multi-aspect ontologies. The approach Proposed by Ruan et al.^17^ uses a metagraph and attention mechanism to capture relationships in the Traditional Chinese Medicine (TCM) clinical data^3^. SaGCN was evaluated using three real-world TCM medical datasets for prediction and diagnostic tasks. This model will be helpful for clinical research and practice. Future studies should incorporate a more diverse range of clinical information. These methods improve diagnosis to some extent but fail to comprehensively explain how regularities are generated using multiple relations among different TCM entities. Challenges include the random structure and poor organisation of TCM clinical prescription data. The machine learning-based approach for cardiovascular disease prediction proposed by Lyu^18^ provides a comparative performance of machine learning models in cardiovascular disease prediction. It was also found that the Random Forest (CART) model had the best performance among the eight models. The Future scope includes improvements in prediction accuracy using ensemble machine learning methods. It discusses the importance of early detection and the challenges of traditional diagnosis. The experiment aimed to identify the best algorithm for building an alert system.

The novel transfer learning model proposed by Liu et al.^19^ generates TCM prescriptions using unstructured resources and limited medical data. It can efficiently learn TCM principles and the interactions between herbs and their symptoms. The model can be used for various TCM tasks, such as syndrome and disease diagnosis. This model can be extended to Western medical tasks if the necessary documentary resources are available. It faces the inability to make prescriptions automatically and static effectiveness of rules. They rely on labelled clinical medical records or images that are usually unavailable^20^. Paul and Anand^21^ presented a new family of similarity measures for scoring confidence in protein interactions. These new measures effectively distinguished true interactions from false positives. These measures may be useful for functional protein clustering analysis. These measures show consistent performance in the biological processes. The evaluation demonstrated that these measures were not limited to any ontology. The ML models advanced by Li et al.^22^ improved the Atherosclerotic Cardiovascular Disease (ASCVD) prediction compared to the PCE (Pooled Cohort Equations) risk calculator. These ML models reduce the number of screenings required to predict the ASCVD risk. The combination of longitudinal and cross-sectional features improves ASCVD prediction. The random forest ML technique (RF) applied to the combination of Cross-Sectional (CS) and Longitudinal (LT) features (RF-LTC) produced the highest AUC score. ML models have higher PPV and lower SCP Sensitivity than the PCE score. However, it also has drawbacks owing to the limited availability of CAC and CCTA data in the study cohort. The Study only included structured EMR data, not signs and symptoms. These Models were not validated in an independent cohort. Potential information bias due to the inclusion of the PCE score. Running models without PCE features did not significantly reduce the accuracy. The methodology suggested by Buoncompagni et al.^23^ includes the development of a model called Arianna, which is a smart home framework for recognising and classifying humans. It uses ontologies to classify sensory data based on contextual knowledge. Arianna manages knowledge based on multiple contexts to evaluate the activity models. This decreases the computational load and ensures the intelligibility. This allows for modular and flexible systems and iterative development processes. However, the use of multiple interconnected ontologies can reduce the reasoning load and improve recognition.

The model presented by Mirzadeh et al.^24^ identified clinical and psychosocial factors that reduce medication nonadherence. The model achieved an average error rate of 12.9%. The present study revealed ten significant factors that predicted medication adherence. These factors include encouragement to play an active role, confidence in emergency situations, knowledge of medications, and having a special person in their lives. The customised transformer-based models initiated by Lentzen et al.^25^ can effectively analyse the structured EHR data. They developed ML-based risk models based on patients’ medical histories. The Ex Med-BERT model was pretrained using MLM and PLOS objectives. The integration of quantitative clinical data improved the prediction performance. Transfer learning allows the leveraging of pretrained models for different clinical endpoints. The IPTW approach was used to adjust for age and sex. The tBNA-PR model was developed by Liang and Guo^26^ for heart failure prediction and patient stratification. The tBNA-PR effectively models temporal EHR data for accurate prediction. This model achieved a prediction accuracy of 0.78 and an F1-Score of 0.7671. Three distinct heart failure subphenotypes were identified. The model provides better quality healthcare services and clinical decision-making support. Li and Zheng^27^ proposed the ChatGPT algorithm to analyse and summarise medical cases in the Song Dynasty literature. AI and prescription recognition technologies have been introduced into medical data mining. The results indicate that the machine classifier performed well in classifying the different prescriptions. Data mining revealed differences in disease stage and drug use. Warm drugs accounted for 45.46% of the phlegm treatments. ChatGPT can be used as an auxiliary tool in biomedical text analysis and clinical decision support^22,28–43^. Ancient medical prescriptions can be better identified and analysed using case data. This supports ancient medical studies and modern medical data warehouses. However, some drawbacks have been observed in the automated case analysis and induction step, leading to an inadequate number of positive samples in the testing dataset, thus impacting the performance of the SVM classifier.

Understanding graph interpretation and healthcare suggestions projects profit from each other. Zhang et al.^44^ proposed the MedRec recommendation model which is a solution for medication suggestions based on limited information. It incorporates a health-related expertise hierarchy and a pharmaceutical attribute chart. This model is considered superior to the latest medical techniques. The two tasks involved in this are graph embedding learning and medication recommendation, which have a strong correlation and mutual benefit, both of which were outperformed by Feng et al.^45^, who suggested the DL method, PhenoBert. When dealing with difficult recognition problems, PhenoBERT performs better and employs a two-level CNN module to increase computational efficiency. If explicit keyword identification is unsuccessful, it may assign ancestor hpo words, which would facilitate the evaluation of health-related textual data. PhenoBERT uses concept identification and text segmentation. In its workflow, PhenoBert performed the best among the three deep learning-based techniques, Phenotagger and NeuralCR. Phenobert also exhibited superior precision and recall, and the imbalance issue in DDI prediction was successfully addressed by the REST model proposed by Li et al.^28^. It is effective for benchmarking DrugBank datasets and selecting suitable samples for the student model using reinforced sampling. This method offers a thorough examination and contrast with alternative balancing techniques. The methods primarily measure the similarity of various features to the predictions. The authors Fafalios et al.^46^ method involves building and utilising the SeaLiT Ontology to store information on maritime history. This makes evidence-based knowledge integration and a common understanding easier to achieve. This integration provides a standard and expandable semantic foundation for data modelling. Historians assisted in exploring and analysing the integrated data. Future developments may encompass the JSON-LD context, enhanced scope notes, and ontology additions. This study was affected by the drawback of incomplete information, which is common in historical archival research. Missing entity attributes can affect the interpretation of the quantitative analysis results. The approach discussed by Yao et al.^11^ which includes the level extraction of ICD and ATC Codes from the ontology and combines it with the diagnosis treatment pathways of the patients, also considers multiple factors to predict the drugs. This study describes a combination of ontology data and DL concepts to facilitate recommendations. The transformer network is used to create the embeddings for the ICD and ATC level codes, and then LSTM is used for disease-drug pair creation which is finally used for the prediction of drugs^7^.

## Methodology

**Fig. 1:**
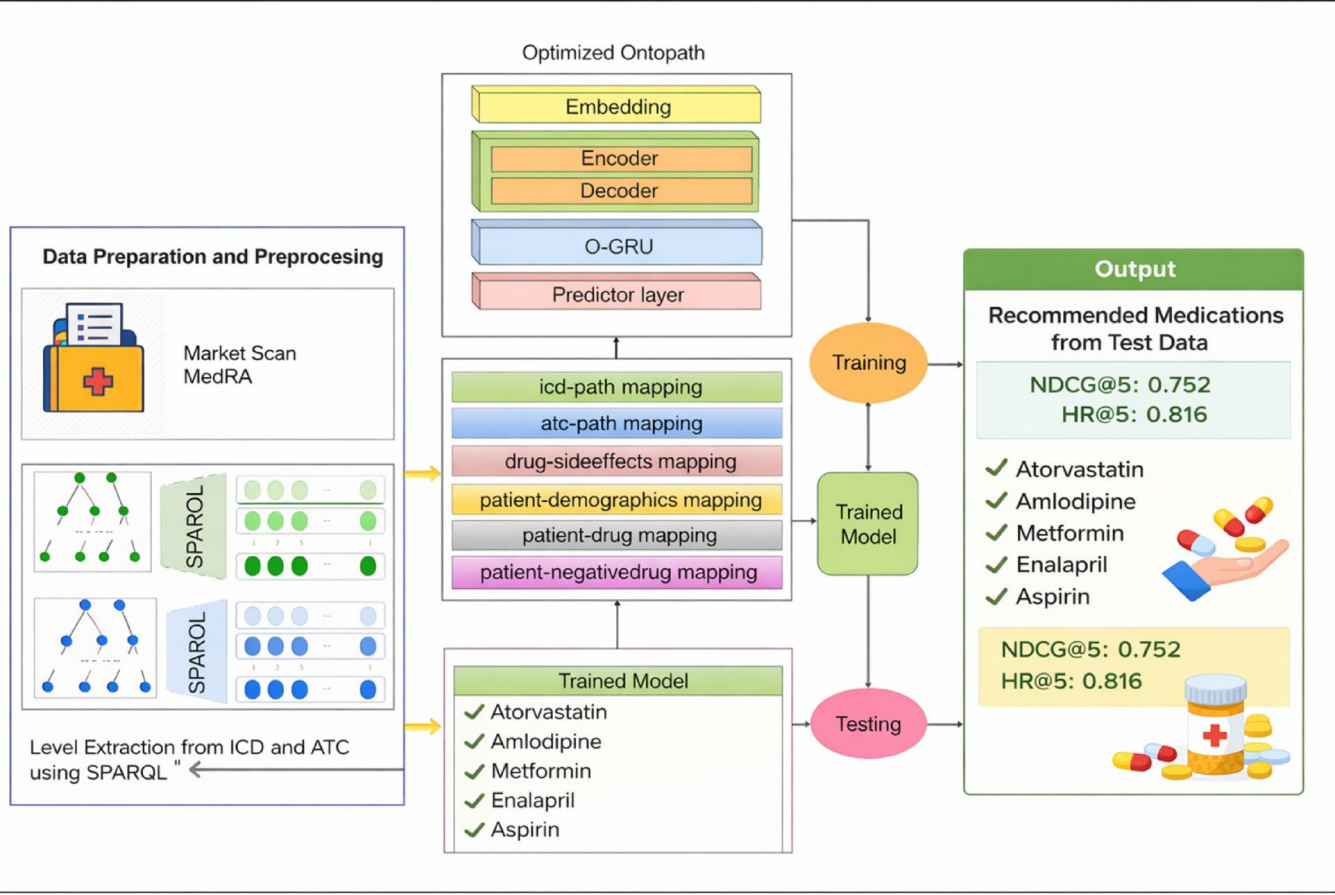
System architecture for deep learning infused ontological framework with E-GRU.

The dataset used in this study includes MarketScan data as Electronic Health Records (EHRs), which provide information on patient demographics, medical history, diagnosis codes, and medications administered during each visit. The cohort was built by selecting 3,000 patients diagnosed with depression, based on ICD disease codes and relevant ATC drug codes. Demographic data such as age, sex, and geographical location were considered to allow for personalized prescription recommendations. The patients in the study are from the MarketScan database, which includes five geographical regions. Information extracted from the dataset includes patient demographics (age, sex, and location), diagnosis codes (ICD-10), prescription data (ATC codes), and side effect information for each drug from the MedDRA database. Additionally, hierarchical data from the ICD and ATC ontologies were extracted to provide a more granular representation of diseases and drugs, aiding in more accurate medication recommendations. In this study, a novel prescription recommendation framework is introduced that incorporates Ontology and DL to predict the next drug in disease treatment pathways. The proposed system involves Preprocessing, Hierarchical Code Extraction, NexusOpti Model Development, Training, Testing and Drug Recommendation. The architecture of the proposed system is shown in Fig. 1. The detailed workflow of each sequential step with the precision of a fine-tuned algorithm is as follows.

### Data preparation and preprocessing

Data preprocessing was performed on structured EHR tabular data, including patient visits, diagnosis codes, and prescriptions. Data preprocessing included the removal of null values, conversion of the attributes to valid data types, and removal of duplicate values. Data preparation involved extracting hierarchical disease and drug information from standardised biomedical ontologies. Specifically, disease hierarchies were extracted from the ICD ontology and drug hierarchies were extracted from the ATC ontology. Hierarchical data refer to multilevel representations of ICD diagnosis codes and ATC drug codes, ranging from coarse-grained categories to fine-grained clinical details. The SPARQL Protocol and RDF Query Language (SPARQL) query OWL-formatted ICD and ATC ontology files for hierarchical data extraction. A disease code was represented using four hierarchical ICD levels, whereas a drug code was represented using five hierarchical ATC levels, which enabled a richer semantic representation of clinical information.

**Fig. 2:**
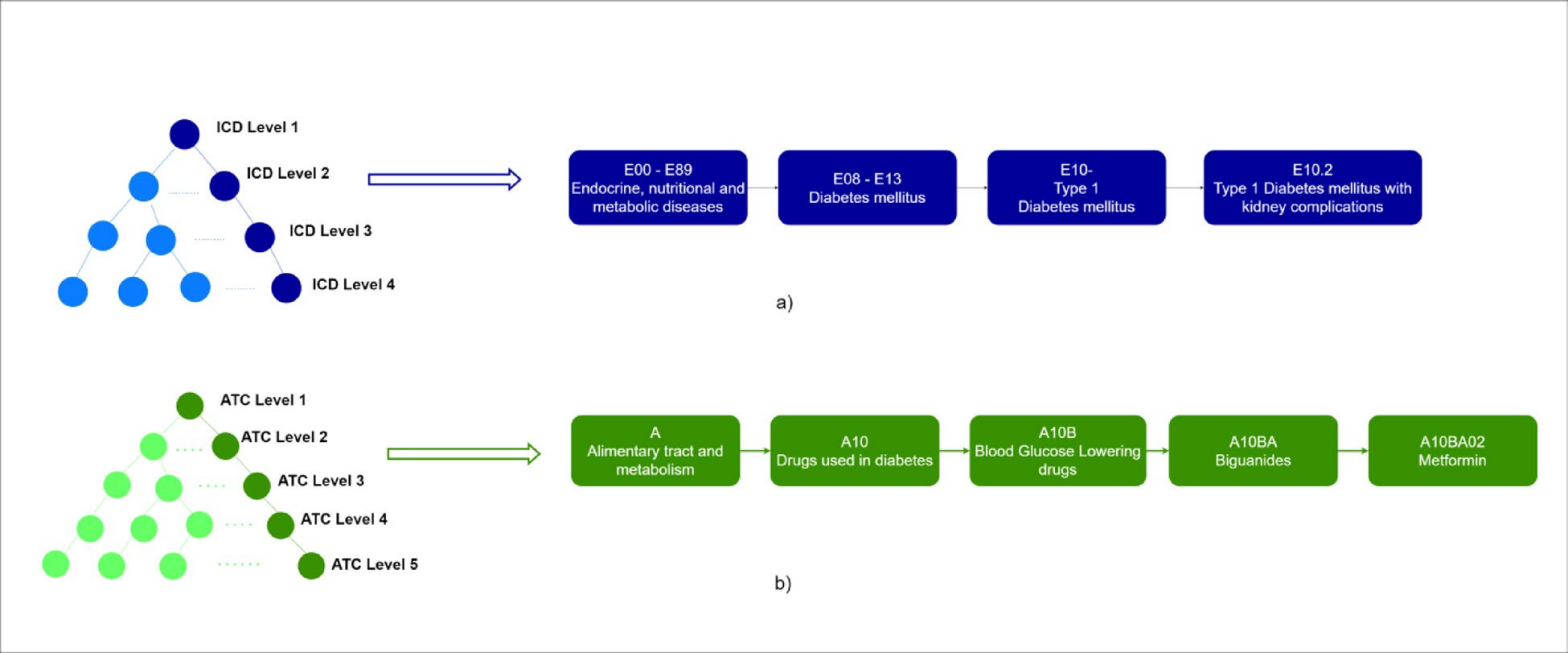
Hierarchical data extraction (**a**) Level extraction from ICD Ontology (**b**) Level extraction from ATC Ontology.

Figure 2 indicates a hierarchical classification of ICD and ATC codes and how ICD code levels were arranged into hierarchical structures, with increasingly detailed information provided at each level. The category, which is a broad classification of connected circumstances, is the first level. The subcategory or second level offers more detailed information regarding a condition. The third-level sub-classification offers more detailed information. Even more narrowly defined, the final level considers the disease even when another condition is present. The detailed classification of the five ATC code levels is presented in Table 1.

**Table 1:** ATC drug classification.

Level	Description	Example
1	Anatomical main group	A – Alimentary tract and metabolism
2	Therapeutic subgroup	A10 – Drugs used in diabetes
3	Pharmacological subgroup	A10B – Blood glucose lowering drugs
4	Chemical subgroup	A10BA – Biguanides
5	Chemical substance	A10BA02 – Metformin

Finally, the retrieved level-to-level extracted codes were stored in a comma-separated value (CSV) file. Because the codes are in an alphanumeric format, they are assigned indices and used for processing. A sample SPARQL query is as follows.

**Figure:**
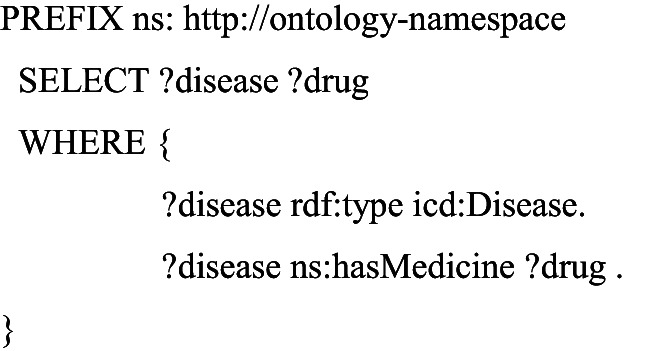

Using SPARQL queries, we hierarchically represented each ICD and ATC code. This hierarchical representation increases the granularity of the data and aids in more precise prescriptions. The algorithm for ICD and ATC level extraction is presented in Algorithm 1.

**Algorithm 1:**
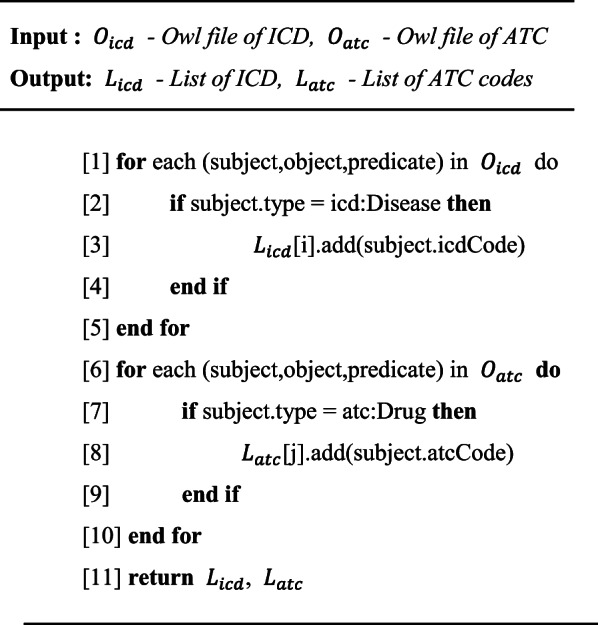
Level to level extraction from ICD and ATC files

Algorithm 1 outlines the procedural steps for generating two distinct lists from the OWL file. Within this algorithm, every subject in the OWL file underwent meticulous examination of the disease codes used in the model. If a subject’s code aligned with the designated ICD code corresponding to that subject, it was methodically appended to the respective list. Similarly, by employing a comparable degree of formality to the ATC codes, the outcome yields two nested lists of terms. Each nested list intricately represents a hierarchical extraction of the ICD and ATC codes. For easier processing, these lists were transformed into CSV files.

### Ethics statement

As this study did not involve human participants, human tissue, or biological samples, institutional ethical approval was not mandatory. Nevertheless, the work complied with the applicable biosafety and organizational data governance requirements and was reviewed and approved by the INTI Biosafety Committee under the Code of Conduct, Section B: Company Information, Records and Assets (8.0 Data Integrity and Data Protection).

### Trainset and testset creation

After the data preparation module, the data were separated into a training set and a test set to make them available for training and testing of the model. To better understand the complex nature of clinical interactions, a series of relational models were developed by integrating processed EHR data into a single relational model, along with an ontology to produce accepted and validated definition sets for the relationships between patients, diseases, medications, and their layered representations. Each disease diagnosis is represented using ICD codes and mapped to its corresponding hierarchical disease path within the ICD taxonomy, thus forming a mapping between the disease and disease path.

Similarly, each ATC code was mapped to its associated drug path within the ATC hierarchy to create a drug-to-drug path mapping^47^. These hierarchical drug representations were derived from the ATC ontology, allowing the model to capture pharmacological similarities among medications. Patient-level contextual information was incorporated by linking patient identifiers to demographic attributes, including age and sex. These patient demographic mappings provide additional context that influences prescription decisions. To account for drug safety considerations, drug–side effect relationships were constructed by associating each drug with its reported adverse effects using unique drug identifiers. This information supports safer medication recommendations by enabling the model to learn the drug risk profiles.

Observed clinical prescriptions are modelled through patient–drug interactions, which represent drugs prescribed to patients during clinical encounters and are treated as positive training samples. In addition, to support discriminative learning, patient–negative drug interactions are generated via a negative sampling strategy, where drugs not prescribed to a given patient are sampled from the global drug set and treated as negative instances during model training. These mappings were created by performing relational joins and index-based lookups across the patient visit records, ICD diagnosis table, ATC prescription table, and ontology-derived hierarchical path table. For ICD and ATC codes, each code was mapped to obtain a unique numerical index, which retrieved the corresponding hierarchical path. Patient–disease and patient–drug relationships are built based on visit-level co-occurrence in the EHR data, whereas negative drug samples are created by excluding the prescribed drugs for each patient.

In the training process, all formed relationships were used for comprehensive learning of the model, whereas in testing, it was ensured that there was an unbiased assessment of the performance of recommendations, thus retaining only the disease-disease path, drug-drug path, patient-demographic, and drug-side-effect relationships. Better performance was achieved with a higher amount of training data; hence, it was advisable to split the data into training and testing sets at a ratio of 80:20 for the model training. The data to be processed must be of the same size as the input data. To ensure this, the data were divided into batches. The batch size was determined during the parameter initialisation. The flow of processing the training and testing data is presented in Algorithm 2. Algorithm 2 frames the creation of the training and testing sets, along with additional mappings. Patient demographic mappings were created from the patient data and demographic information with reference to the patient id, patient-demographics mappings are created. Similarly, patient-side effect mappings were created for the drug ID. Similarly, icd-path and atc-path mappings were created.

**Algorithm 2:**
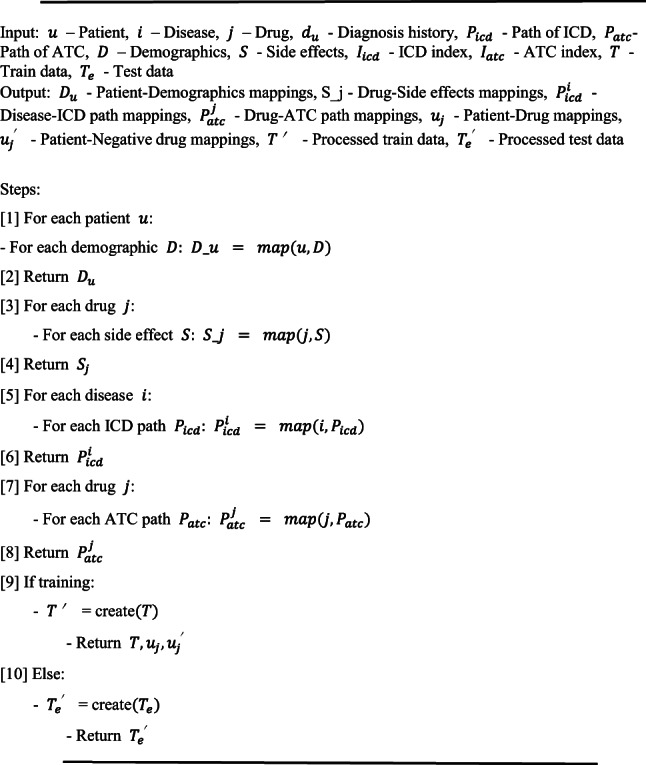
Mappings creation for Trainset and Testset

### NexusOpti model construction

The proposed NexusOpti model is a deep learning (DL) neural network model. A neural network is inspired by the computation of the human brain, which works through neural units called neurons. The input, hidden, and output layers comprise a neural network. Computations are performed at the hidden layers, and as the number of hidden layers increases, the learning rate of the model also increases, resulting in excellent performance. Therefore, the proposed model includes several layers. The NexusOpti model was developed using four layers: The layers included were embedding, transformer, Enhanced GRU, and predictor layers. The implementation of these layers is explained in detail in the following sections.

#### Embedding layer

A two-dimensional array encased in a module container with functionality is called an embedding. It uses the number of embeddings and dimensions of each embedding as inputs. Two embedding layers, one for processing the ICD and the other for the ATC, were created. Although clinical data may contain variations in terminology, this study relied on standardised ICD and ATC codes, which are already normalised representations used in administrative EHR systems. As a result, the clinical diagnosis and prescription records directly aligned with the concepts defined in the ICD and ATC ontologies, eliminating lexical mismatch issues commonly observed in free-text clinical notes. The size of the ICD embedding was [41334, 64], and for ATC embedding, the size was [6552,64].

1 $$\left[ {\begin{array}{*{20}l} {P_{1} } \hfill & {P_{2} } \hfill & \cdots \hfill & {P_{n} } \hfill \\ \end{array} } \right] = \begin{array}{*{20}c} {d_{11} } & {d_{21} } & \cdots & {d_{T1} } \\ {d_{12} } & {d_{22} } & \cdots & {d_{T2} } \\ {d_{13} } & {d_{23} } & \cdots & {d_{T3} } \\ {d_{14} } & {d_{24} } & \cdots & {d_{T4} } \\ \end{array}$$

Equation (1) represents how the hierarchical data extracted from the OWL file are used as matrix. Here, $d_{tl}$ denotes the Level path for ICD/ATC codes diagnosed during visit, *t* and *l* is the level of the code in the Level path. The Cumulative Level path for a patient at *i*th visit is denoted as $P_{i}$, where i is the visit of the patient. The workflow of the embedding layer in the model is presented in Algorithm 3 below.

**Algorithm 3:**
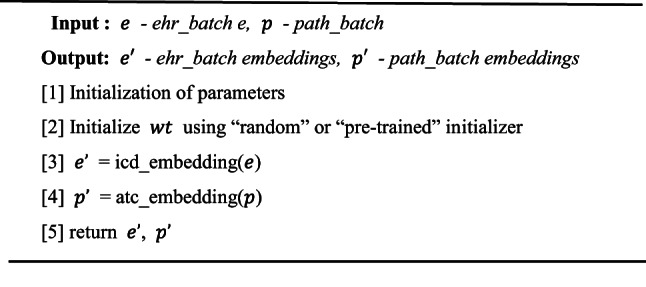
Embedding Creation

ehr_batch refers to the icd_sequence which consists of the codes that represent the diagnosis history of the patient, and path_batch refers to the level-to-level path of the drug codes recommended to the patient.

#### Transformer layer

The transformer layer consists of encoder and decoder layers. used the levels extracted above to form a level path for each diagnosed code. Subsequently, for each row of the hierarchically represented matrix, we added position information to the data sequences separately for odd and even positions. Thus, an input embedding matrix was generated. Equations (2) and (3) explains how the position information is added.

2 $$POS_{t,2l} = \sin \left( {\frac{t}{{10000^{\frac{2l}{K}} }}} \right)$$

3 $$POS_{t,2l + 1} = \cos \left( {\frac{t}{{10000^{\frac{2l}{K}} }}} \right)$$

where *K* denotes the dimension of the data, *l* is the index in, *K* and *t* are the time steps in the diagnostic history. Next, multi-head self-attention is implemented to encode the resultant data after adding position information. To do so, a query embedding, key embedding, and value embedding are computed for the input embedding *I*. After computing, self-attention for a single head is generated using Eq. (4).

4 $$E_{n}^{\left( I \right)} = softmax\left( {\frac{{Qry_{n}^{\left( I \right)} Ky_{n}^{\left( I \right)} }}{\sqrt d }} \right)Val_{n}^{\left( I \right)}$$

Here, $E_{n}^{\left( I \right)}$ implies the final encoded results. $Qry_{n}^{\left( I \right)}$, $Ky_{n}^{\left( I \right)}$, $Val_{n}^{\left( I \right)}$ are the query embedding, key embedding, and value embedding of *I* respectively. For the multiple-head self-attention, a multilayer perceptron (MLP) function was used to obtain the overall encoded result, as shown in Eq. (5).

5 $$E^{\left( I \right)} = MLP\left( {concat\left( {E_{1}^{\left( I \right)} , \ldots ,E_{n}^{\left( I \right)} } \right)} \right)$$

Next, the encoded data were decoded using patient demographic data. To accomplish this, the patient demographic vector was mapped using a query embedding. Similar to the encoder process, the query, key, and value matrices are computed, and decoding is performed using Eq. (6).

6 $$D_{z,n}^{\left( u \right)} = softmax\left( {\frac{{qry_{n}^{\left( o \right)} Ky_{n}^{\left( E \right)} }}{\sqrt d }} \right)Val_{n}^{\left( E \right)}$$

$D_{z,n}^{\left( u \right)}$ is the resultant decoded patient embedding, $qry_{n}^{\left( o \right)}$ is the query embedding obtained using patient’s demographic vector, $Ky_{n}^{\left( R \right)}$ is the key embedding of , $Val_{n}^{\left( R \right)}$ denotes the Value embedding of $E_{n}^{\left( I \right)}$. Similar to the encoder for the multi-head attention, Eq. (5) is applied to the decoded matrix. The pseudocode for this layer is presented in Algorithm 4, as follows:

**Algorithm 4:**
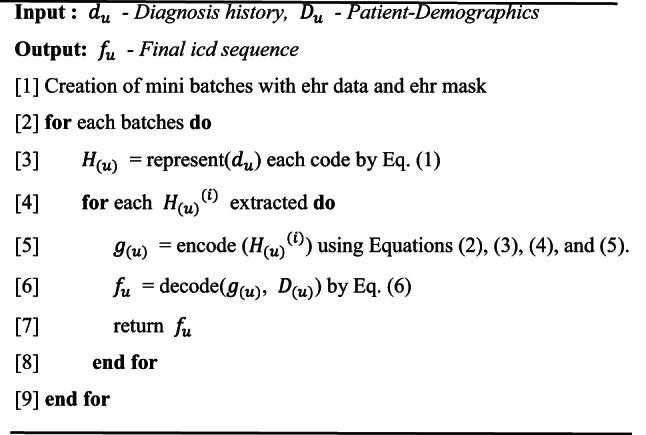
Transformer Layer

#### E-GRU layer

**Fig. 3:**
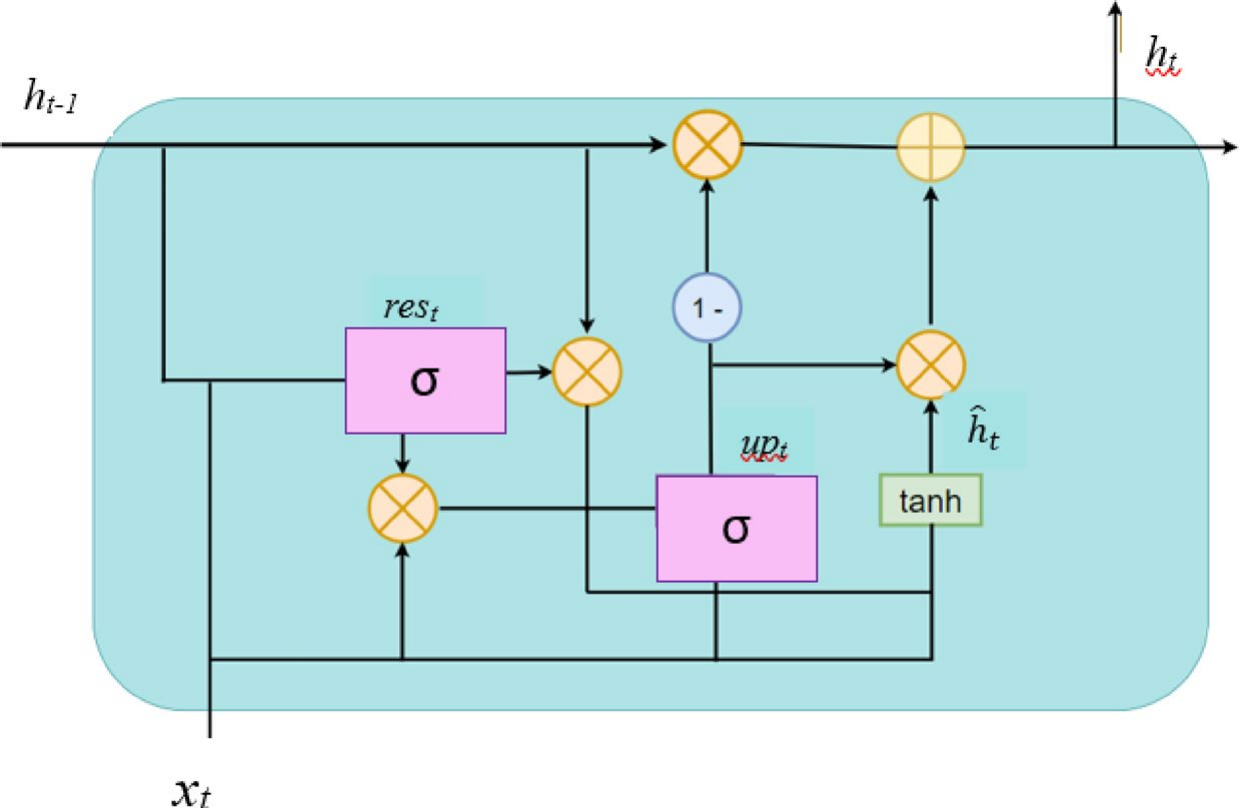
Architecture of E-GRU illustrates the different components of the E-GRU. The color coding used to identify and differentiate the components in the diagram. The pink boxes represent activation sigmoid functions (reset gate and update gate), the yellow circles show element-wise multiplication used for gating, and the green box represent the ‘tanh’ activation function.

An Enhanced GRU Layer was implemented, and the gate output was used to filter the data to remove redundant information from the current input. The reset gate modifies the output of the update gate, which can hasten convergence and decrease gradient attenuation, thereby increasing learning efficiency. Figure 3 illustrates the modified GRU, that is, the architecture of the Enhanced GRU.

The mathematical representations of the operation of the two gates, reset gate, and update gate, present in the E-GRU, are explained by Eqs. (7) and (8), respectively. Equation (9) representing the candidate hidden state after applying the ‘tanh’ activation. Final output state is denoted by Eq. (10).

7 $$res_{t} = \sigma \left( {x_{t} \otimes WG_{1r} \oplus h_{t - 1} \otimes WG_{2r} } \right)$$

8 $$up_{t} = \sigma \left( {x_{t} \otimes WG_{1u} \otimes res_{t} \oplus h_{t - 1} \otimes WG_{2u} } \right)$$

9 $$\hat{h}_{t} = \tanh \left( {x_{t} \otimes WG_{1g} \oplus \left( {res_{t} \oplus h_{t - 1} } \right) \otimes WG_{2g} } \right)$$

10 $$h_{t} = \left( {\left( {1 - up_{t} } \right) \otimes \left( { h_{t - 1} } \right) + up_{t} \oplus \hat{h}_{t} } \right)$$

Here $res_{t}$ is the reset gate, $up_{t}$ denotes the update gate, hidden state is denoted by $h_{t}$, $x_{t}$ is the input at time t, $WG_{1}$ and $WG_{2}$ are weight matrices, $\hat{h}\_t$ is candidate hidden state, and $h_{t - 1}$ is the hidden the state at previous loop. After computing the candidate hidden state, the final output state is weighted combination of the previous hidden state $\left( {{\boldsymbol{h}}_{{{\boldsymbol{t}} - 1}} } \right)$ and the candidate hidden state $\left( {\hat{h}\_t} \right)$, as indicated by the summation and “1-” operation in the diagram. At each time step, the E-GRU computes a candidate hidden state (ht_candidate). After processing all time steps, the final hidden state (ht_final) is computed as a weighted combination of all previous candidate states (ht_candidate) and the previous hidden state (ht-1). This final hidden state is then passed to the hierarchical prediction layer.

**Algorithm 5:**
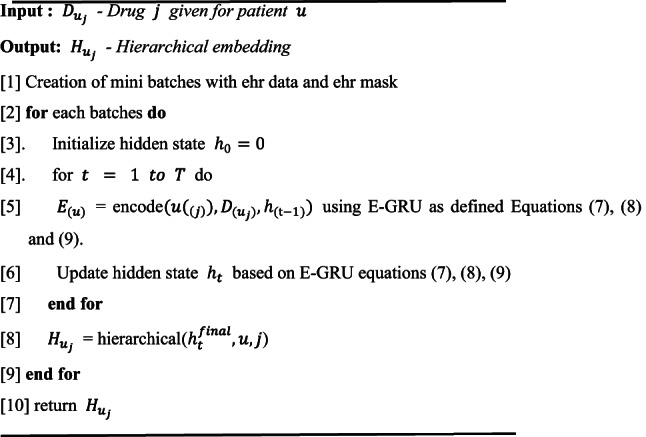
Enhanced GRU Layer

The final hidden state (ht_final) produced by the Enhanced GRU (E-GRU) layer is passed as input to the hierarchical prediction layer, where it is used to predict the next drug in the treatment pathway. This final hidden state is a weighted combination of all previous hidden states, representing the most relevant information learned from the patient’s medical history. The hidden layers were composed of GRU neurons. The input data of the GRU neural network were the data at time t after data preprocessing. The input data were time-series data. The traditional GRU model has the disadvantages of low update efficiency and poor information processing capability. GRU has problems, such as a slow convergence rate and low learning efficiency. The E-GRU model improves the information processing capability and learning efficiency by optimising the unit structure and learning mechanism of the GRU. E-GRU uses a reset gate to optimise the learning mechanism of the GRU, thereby improving the learning efficiency and prediction performance.

#### Prediction layer

The hierarchical prediction layer takes as input the final hidden state (ht_final) from the E-GRU layer. This state is then passed through fully connected layers to map the learned representations to drug recommendations. The output of this layer is the predicted list of drugs, ranked according to their relevance to the patient’s current disease and medical profile. In this layer, the final prediction score was computed using two predictors, MLP and DOT, and two prediction activation functions, ELU and SiLU. The defined predictor layer had a size of [1, 128] and recommendation score ranges from zero to one.

### Training

The model is fed with the dataset during the training phase so that it can obtain as much knowledge as possible regarding the types of data it will be analysing. Embedded patient, drug, and side effect data were provided to the model for learning.

#### Pretraining

The pretraining of a neural network is essentially the process of training a model for a single task or dataset. Subsequently, a new model is trained on a distinct task or dataset using the parameters or model from this training. Thus, the model has an advantage over starting from scratch. During pretraining, the level of aggregated embeddings of the patient and drugs was given. The discriminativeness of the embeddings was pretrained using Contrastive Learning. The contrastive loss is calculated using Eq. (11).

11 $$Loss_{pretrain} = \frac{1}{B}\mathop \sum \limits_{n = 1}^{B} \log \left( {\frac{{\exp \left( {\frac{{sim\left( {e_{n}^{\left( u \right)} ,e_{n}^{\left( d \right)} } \right)}}{\tau }} \right)}}{{\exp \left( {\frac{{sim\left( {e_{n}^{\left( u \right)} ,e_{k}^{\left( d \right)} } \right)}}{\tau }} \right)}}} \right)$$

$Loss_{pretrain}$ denotes the Contrastive loss for pretraining, *B* is the Batch size used for processing, *sim* defines the similarity function which can be either cosine similarity or dot product, *τ* is the hyperparameter for softmax temperature, $e_{n}^{\left( u \right)}$ contains the level aggregated embedding for patient u and $e_{n}^{\left( d \right)}$ has the level aggregated embedding drug *d*. Negative sampling was performed to train the model using incorrect data. If we have one sample, then there will be *B - 1* negative samples.

### Testing and drug recommendation

In the testing phase, a final recommendation label was first generated, and the recommendation score for each drug was then computed. The output is a recommendation score matrix ${\mathrm{M}} \in { \mathbb{R}}^{U \times D}$, where *U* denotes the number of patients in the test set and *D* represents the total number of distinct drugs. Each entry $M_{u,d} \in \left[ {0,1} \right]$ reflects the predicted probability of a drug being *d* is suitable for the patient *u*. In this study, the candidate set comprised 6,552 ATC-classified drugs, for which the model computed the recommendation scores per patient. The final recommendation label is found using the Eq. (12)

12 $$L^{u,i} = \sigma \left( {w^{{\left( {out} \right)T}} concat\left( {\left( {e^{\left( u \right)} \odot s^{\left( d \right)} } \right),\left( {s^{\left( u \right)} \odot se^{\left( d \right)} } \right)} \right)} \right)$$

Here, $e^{\left( u \right)}$ is the level-aggregated embedding patient *u*, $e^{\left( d \right)}$ has the level-aggregated embedding drug *d*, $se^{\left( i \right)}$ is the level-aggregated embedding for the side effect of drug *d*, $L^{u,d}$ shows the classification label which can be either 1 or 0, and denotes the output parameters.

We then used cross-entropy to optimise the positive and negative sampled drug pairs. The cross-entropy was computed using Eq. (13).

13 $$Loss_{entropy} = - \sum\limits_{{\left( {u,d} \right)}} {\alpha \log \alpha + \left( {1 - \alpha } \right)\log \left( {1 - \alpha } \right)}$$

where $Loss_{entropy}$ denotes the cross-entropy loss, $\alpha = L^{{\left( {u,d} \right)}}$ has the classification label. In this module, the recommendation score for each drug was determined. The drug scores ranged from zero to one. The score for drugs labelled 1 was greater than 0.5. This output formulation enables ranking-based evaluation and facilitates performance assessment using standard metrics, including the Hit Ratio and NDCG. The operations of the training and testing modules are described in Algorithm 6.

**Algorithm 6:**
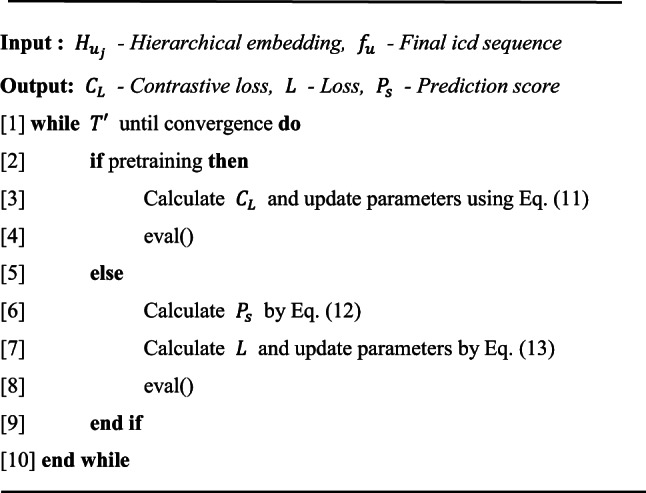
Training and Testing

## Experimentation

### Dataset

Standard ICD and ATC Ontologies were used for the acquisition of ICD and ATC codes, respectively. The extracted ICD Codes belonged to version 10. A detailed description of the ICD and ATC ontology files is presented in Table 2.

**Table 2:** ICD and ATC description.

Ontology	Properties	Count
ICD Ontology	Number of classes	22,533
	Properties	7
	Maximum depth	7
	Maximum number of children	21
	Classes with single child	38
	Number of Classes	6,799
ATC Ontology	Properties	3
	Maximum depth	7
	Maximum number of children	47
	Classes with single child	207

Since the ICD has a huge disease database, the study narrowed our scope to depression and MarketScan data were used as EHRs. It contains information on patient demographics, medical history, diagnosis codes, and medications administered at every visit. Age, sex, geographical position, and health status were included in the demographic file. ICD codes, indexed for easier processing, represent diagnosed diseases. ATC codes were indexed for ease of processing. Because negative sampling was involved, we used a label to denote depression and non-depression. For negatively sampled pairs, the label was set to 0, and the others were set to 1.

**Fig. 4:**
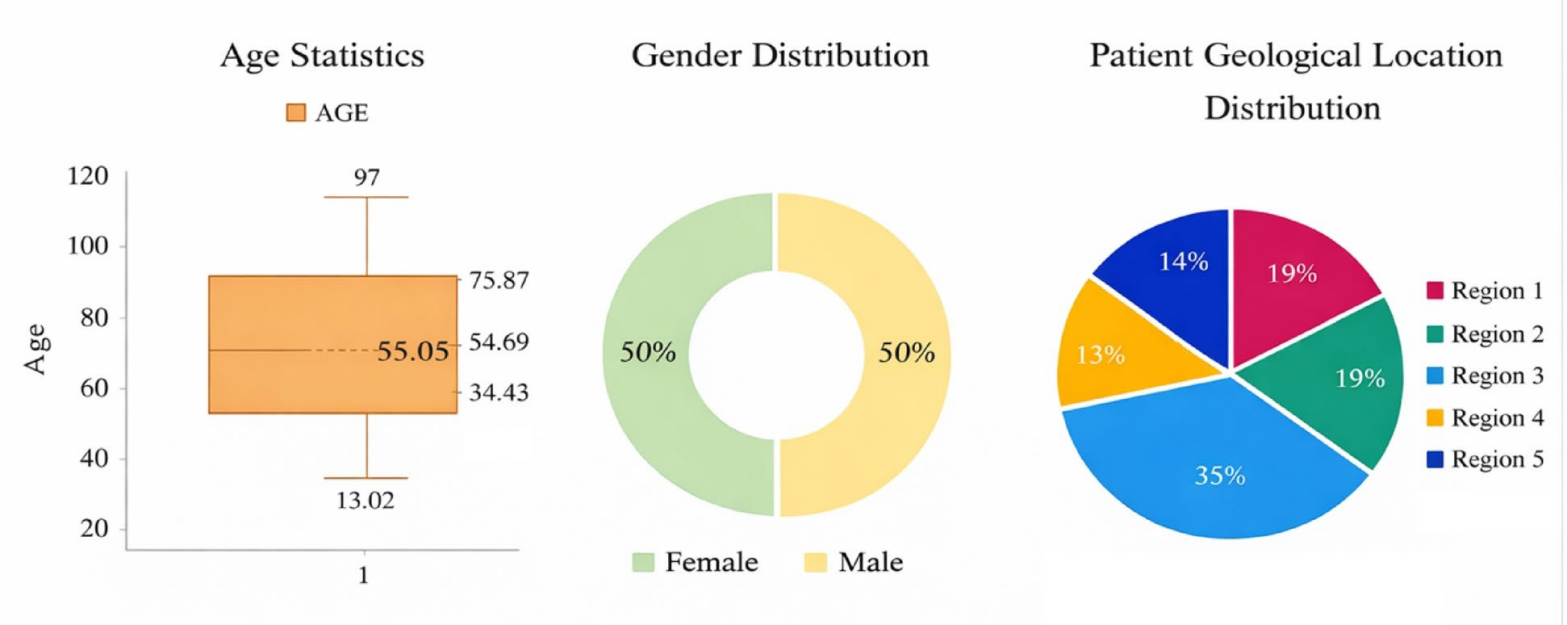
Demographics information of patients.

This study included a cohort of 3000 patients. In the demographic information of patients, we considered various factors to prescribe more personalised medicine for patients. The factors considered were age, sex, and location. The age is further divided into five sub groups where the categories are *≤ 20*, *≤ 40*, *≤ 60*, *≤ 80* and *≤ 100*. The cohort included 1514 male and 1486 female patients. The geographical locations of the patients were also considered and subdivided into five groups. A patient could belong to any of these five regions, and the demographic distribution of the participants is shown in Fig. 4. Side effect data for each drug were acquired from the Medical Dictionary for Regulatory Activities (MedDRA). It contains a database of the side effects of drugs used in humans. The gold standard was built by integrating expert-reviewed drug-disease mappings from the ICD and ATC ontologies, along with drug-side-effect relationships from the MedDRA database. Positive drug-disease instances were modeled as prescriptions, while negative instances were generated through negative sampling. Expert validation ensured the accuracy and clinical relevance of the recommendations.

**Fig. 5:**
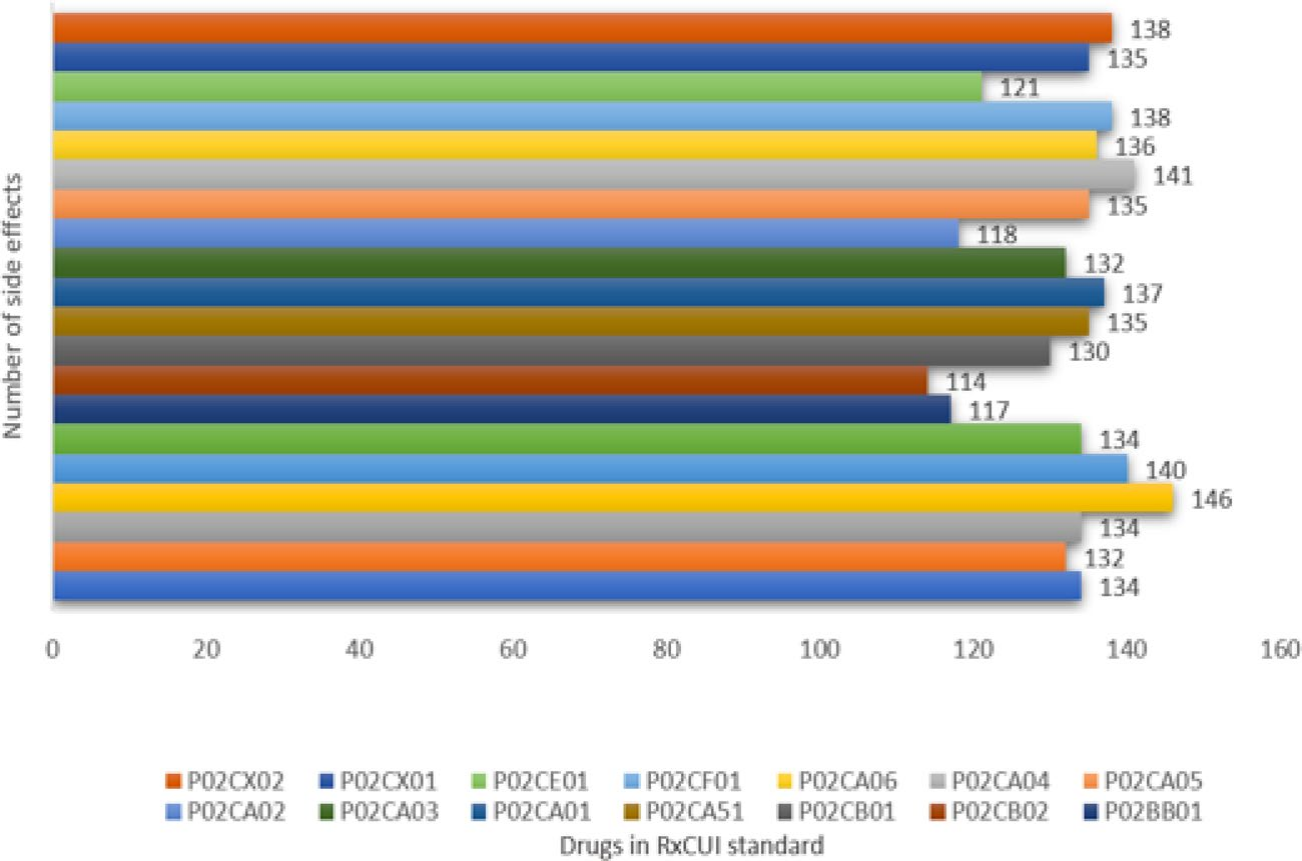
Drug side effects count.

Considering the side effects of all drugs, we created a side effect vector of size 312 to represent the complications of a drug in binary vector form. The drugs and the number of side effects are shown in Fig. 5. This fig. shows the corresponding drug ID and the number of side effects. The drugs in the chart are represented by the RxCUI standard, which refers to a code that uniquely identifies each drug.

### Experimental settings

A Windows operating system with 16 GB RAM and an Intel Core i5 processor were used to develop this model. Python programming language was used for the implementation of the DL model, and SPARQL was used for working with OWL files. The platforms used for the execution and development of the model included Jupyter Notebook and Google Colaboratory. To support both the ontology and DL models, we used the PyTorch framework and libraries such as rdflib, numpy, pandas, and scikit-learn which were installed and imported for use with Jupyter or Colab.

### Evaluation metrics

In the field of information retrieval and recommendation systems, the hit ratio and NDCG are two frequently used assessment metrics. The most recent antidepressant prescription for each patient in the testing set was used as a benchmark. We generated a top-k suggestion list for each patient by prioritising the prescriptions and candidates according to their estimated odds. We then evaluated these recommendations using two metrics and presented the overall performance by averaging the findings for all the tested patients. The performance of the proposed method was evaluated using evaluation metrics such as the hit ratio and the NDCG.

In the case of the Hit Ratio, given the top-k list for antidepressant recommendations, we checked whether the ground truth drug was in the list. If yes, we marked 1 for this patient and 0 for the others. It is mathematically represented in Eq. (14)

14 $$Hit\;Ratio = \frac{{r_{k} }}{R}$$

Here, $r_{k}$ denotes the top *k* relevant recommendations, and *R* contains all relevant recommendations. The next evaluation metric was the normalised discounted cumulative gain (NDCG). Given the top-k list of antidepressant recommendations, we considered the ranking position of the ground-truth drugs in the list. The score decreased as the rank of the ground truth decreased (0 if it was outside the ranking list), as shown in Eq. (15).

15 $$NDCG@k = \frac{{\sum\nolimits_{i = 1}^{{k\left( {actualorder} \right)}} {\frac{Gain}{{\log_{2}^{{\left( {i + 1} \right)}} }}} }}{{\sum\nolimits_{i = 1}^{{k\left( {idealorder} \right)}} {\frac{Gain}{{\log_{2}^{{\left( {i + 1} \right)}} }}} }}$$

The hit ratio is a simple metric that indicates how well a system can return relevant results in response to user requests. Owing to its simplicity, it is highly interpretable and computationally efficient. By considering both the relevance of the retrieved items and their locations in the ranked list, the NDCG provides a more thorough assessment of the retrieval quality. By penalising lower-ranked relevant items more severely than higher-ranked ones, this metric improves upon the hit ratio’s weaknesses and more closely matches users’ desires for relevant things to show higher in the list.

## Results

### Data extraction

As a novel approach in this study, considering the levels of ICD codes and ATC codes has increased granularity, resulting in a more precise prescription. The extracted levels of some ICD codes are presented in Table 3.

**Table 3:** ICD level to level extraction.

ICD code	Level 1	Level 2	Level 3	Level 4
F32.0	F00–F99	F30–F39	F32	F32.0
F32.1	F00–F99	F30–F39	F32	F32.1
F32.2	F00–F99	F30–F39	F32	F32.2
F32.3	F00–F99	0F30–F39	F32	F32.3

Here, F32.0 is the ICD code for major depressive disorder with a single depression episode. It is the most specific code which is a subclass of depressive episodes, represented as F32 in Table 3. F32 is a class in F30-F39, which includes mood-affecting disorders. The generic class F00-F99 which includes all the codes related to mental and behavioural disorders, and the extracted levels for some ATC codes are listed in Table 4.

**Table 4:** ATC level to level extraction.

ATC code	Level 1	Level 2	Level 3	Level 4	Level 5
N06AB03	N	N06	N06A	N06AB	N06AB03
N06AX16	N	N06	N06A	N06AX	N06AX16
N06AF04	N	N06	N06A	N06AF	N06AF04
N06AX27	N	N06	N06A	N06AX	N06AX27

In Table 4, the ATC code “N06AB03” is considered. Here, the first level, represented by the letter "N”, represents the primary anatomical group and shows how the medicine affects the nervous system. Moving on to the next level, “N06”, the pharmaceuticals are classified as psychoanaleptics, which include antidepressants and other treatments that work on the central nervous system to relieve mood problems. Moving on to the third level, “N06A”, which identifies antidepressants and describes the drug’s pharmacological class. This is further classified at the fourth level, "N06AB”, as a subset of antidepressants that impede serotonin reuptake in the brain. Finally, fluoxetine was represented by “N06AB03” at the fifth level. This medication is frequently recommended for treating depression, OCD, bulimia nervosa, and other mental health problems.

### Performance analysis using evaluation metrics

**Fig. 6:**
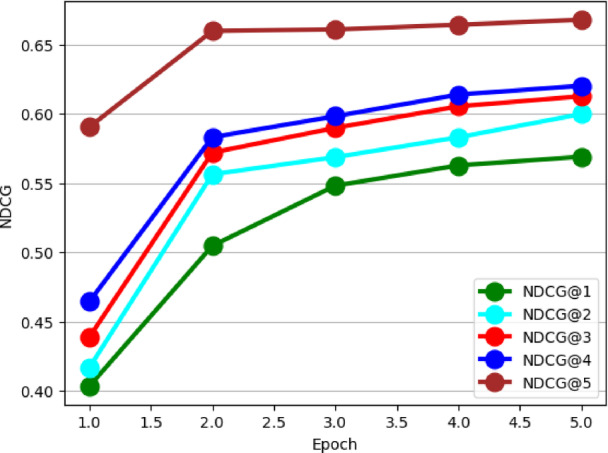
NDCG metrics for Epochs = 5.

The results of the comparison of the evaluation metrics with the implementation of the five epochs at the five levels are visualised in detail. Figure 6 shows the NDCG comparison for five epochs. Each plot shows the NDCG values at five levels.

**Fig. 7:**
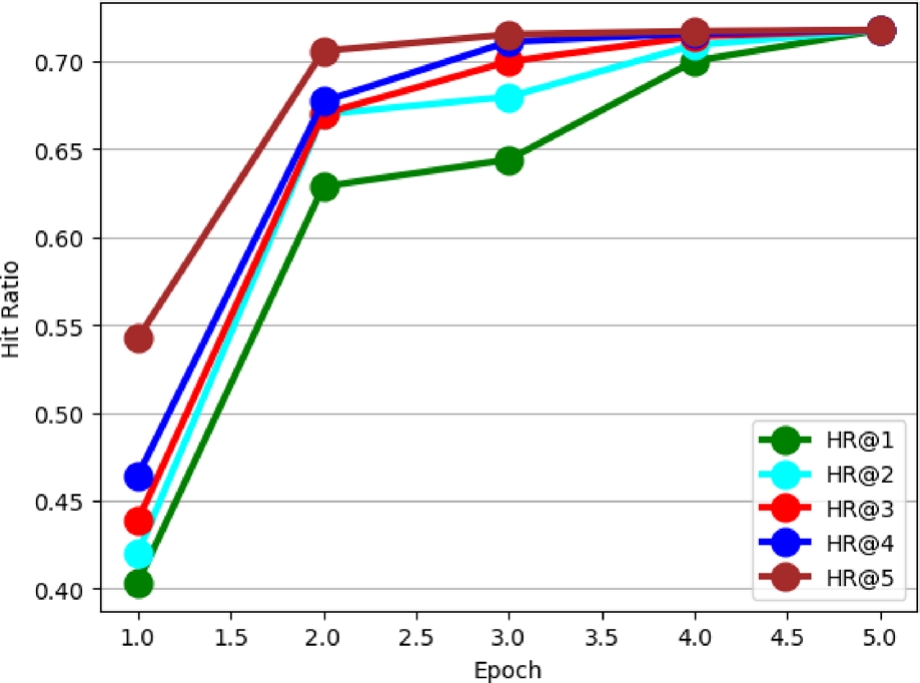
Hit Ratio metrics for Epochs = 5.

A comparison of the hit ratios for the five epochs at the five computation levels is shown in Fig. 7. From the results, it was inferred that at level 5, the maximum hit ratio and NDCG were obtained.

**Fig. 8:**
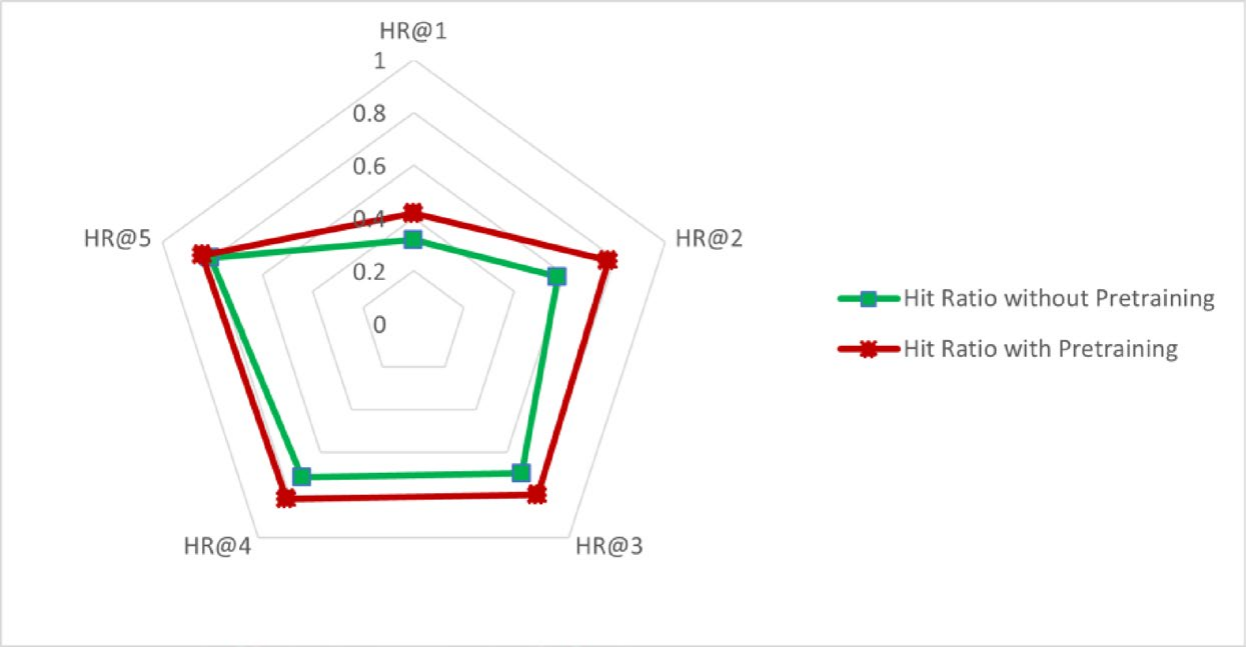
Hit Ratio – pretraining vs non-pretraining.

The model with a comparison of pretrained and non-pretrained models and their differences in the evaluation metrics are shown in Fig. 8, where the pretrained model shows higher performance than the non-pretrained model.

**Fig. 9:**
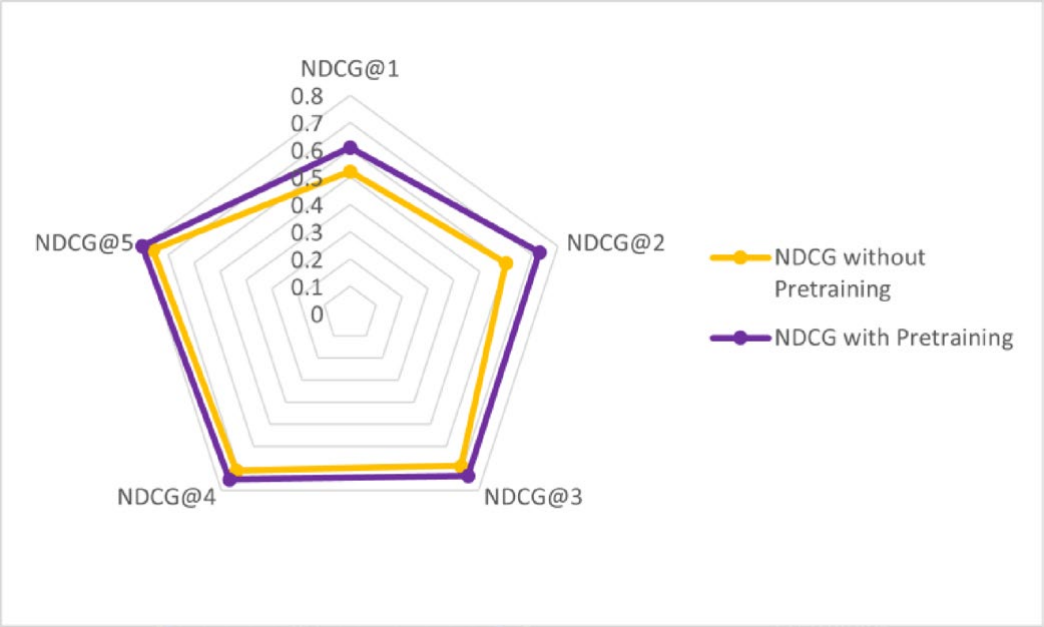
NDCG – pretraining vs non-pretraining.

Figure 9 shows a comparison of the NDCG values for the pretrained and fine-tuned models. From both comparisons, it is clear that the pretrained model outperforms the fine-tuning.

### Performance analysis with variation in dimension embedding

The first comparison involved modifying the dimension of the embedding (dim_emb) to describe the dimensionality of the embedding space used to represent categorical information, including ICD codes, ATC codes, patient demographics, and side effects of the medication. The complexity and richness of the latent representations can be adjusted by changing the dim_emb. While a smaller dim_emb might result in a more compact representation at the expense of some information capture, a higher dim_emb might enable the model to capture more complex interactions between categorical characteristics but also raise computing costs and increase the risk of overfitting. Table 5 presents a wide comparison of the models with embeddings of various dimensions. The hit ratio was used as the evaluation metric for this comparison.

**Table 5:** Hit ratio comparison with variation in dimension embedding.

Embedding dimension (d)	Hit ratio				
	HR@1	HR@2	HR@3	HR@4	HR@5
32	0.413	0.701	0.724	0.758	0.791
64	0.417	0.774	0.798	0.817	0.839
128	0.419	0.799	0.801	0.854	0.873

The same is also compared with respect to NDCG which is listed in Table 6. The Fig. shows that the NDCG for dimension embedding 128 has a higher performance but with a high computation cost and time. Hence, is not preferred over dimension embedding 64.

**Table 6:** NDCG comparison with variation in dimension embedding.

Embedding dimension (d)	NDCG				
	NDCG@1	NDCG@2	NDCG@3	NDCG@4	NDCG@5
32	0.6062	0.692	0.713	0.724	0.727
64	0.6085	0.729	0.7332	0.7396	0.7499
128	0.623	0.735	0.764	0.791	0.812

### Performance analysis with variation in optimizers

**Fig. 10:**
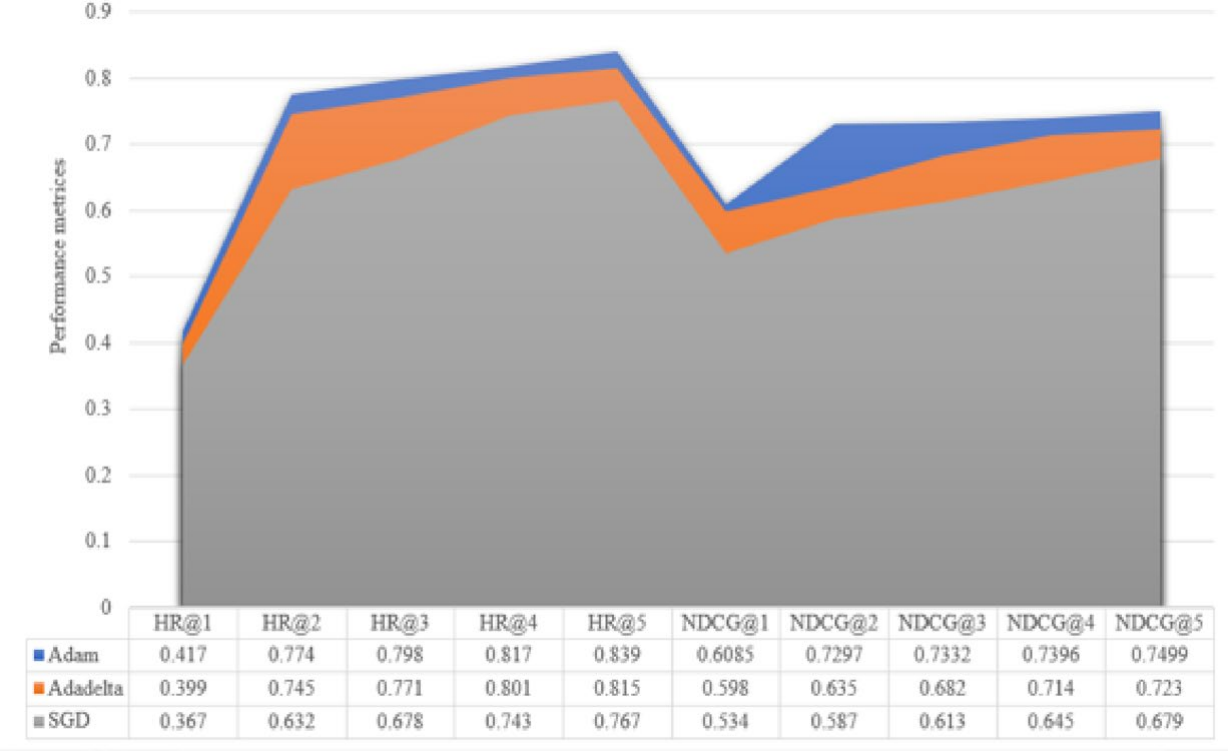
Performance comparison with different optimizers.

Optimiser plays a crucial role in training neural networks by adjusting the model parameters to minimise the loss function during the training process. A performance comparison of different optimisers, such as Adam, Adadelta, and SGD, is shown in Fig. 10. From the visualisation, it is proven that the Adam optimiser has a higher performance than other optimisers.

### Comparison with related works

**Fig. 11:**
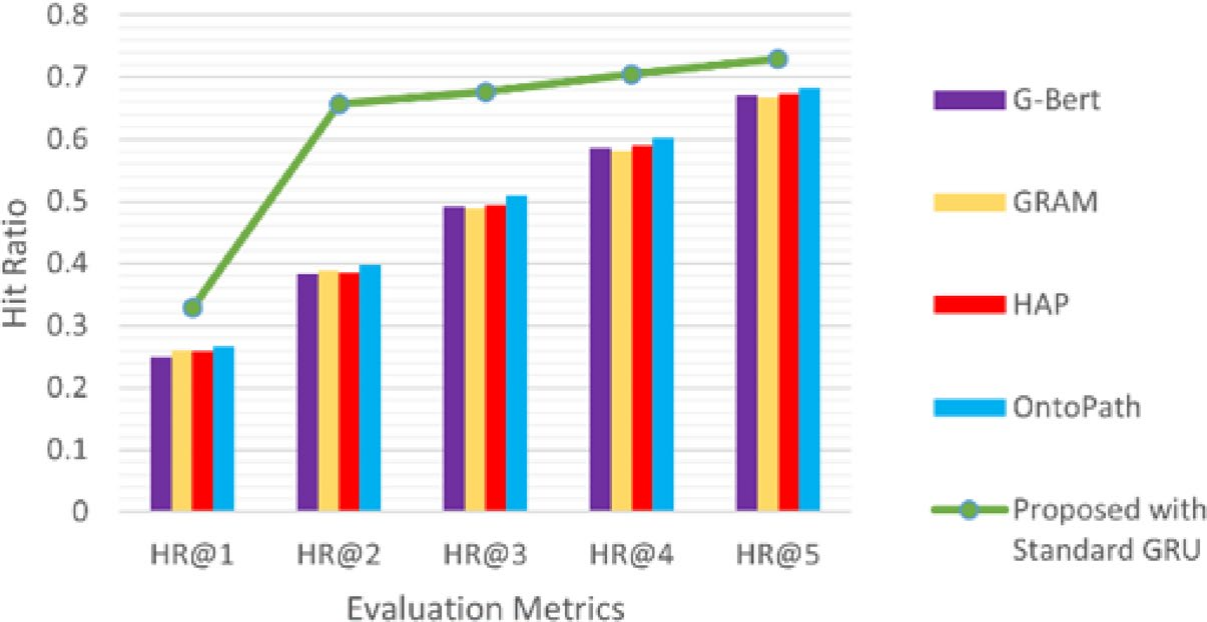
Hit ratio comparison with existing systems.

In this study, the proposed NexusOpti framework was compared with other baseline systems, including GRAM, G-BERT, HAP, and OntoPath. Figure 11 shows a comparison of the baselines with the NexusOpti model and the implementation of NexusOpti with the traditional gated recurrent unit (GRU). Using the GRU, there was a considerable surge in the hit ratio.

**Fig. 12:**
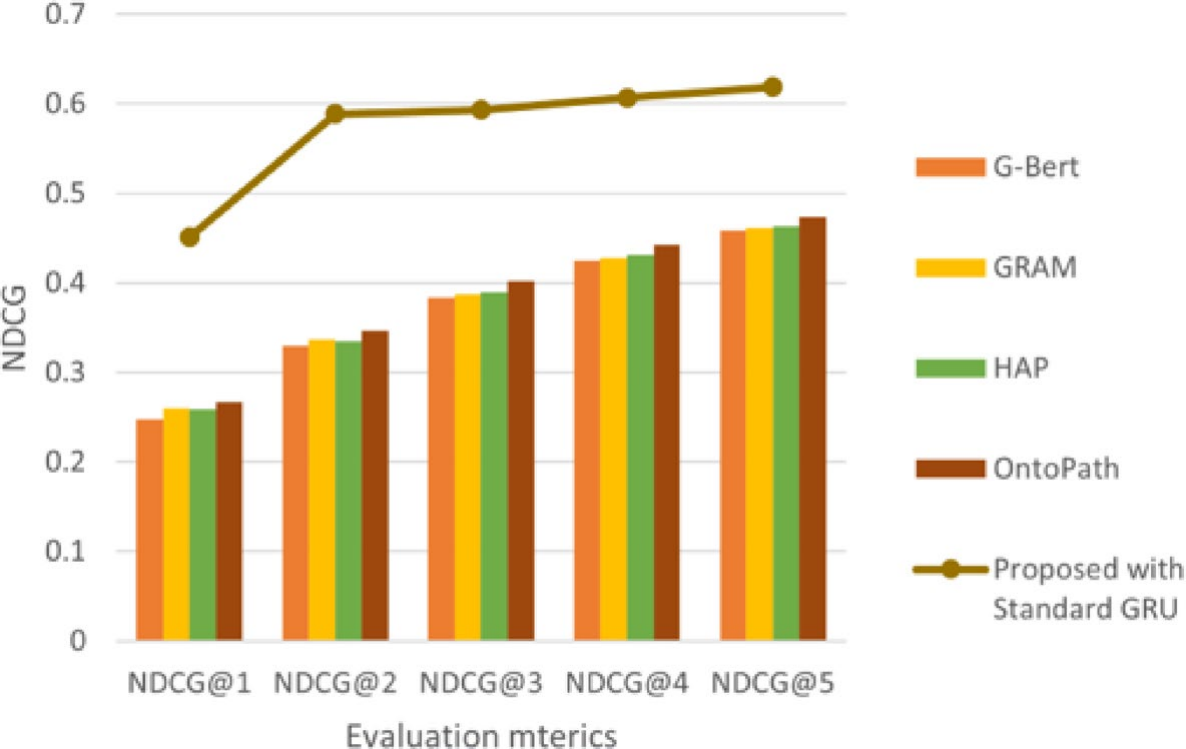
NDCG comparison with existing systems.

The NexusOpti model with the traditional GRU was also evaluated based on the NDCG metric with the existing systems, as shown in Fig. 12.

With our novel E-GRU, which replaces a traditional GRU layer, there was a substantial increase in the overall performance of the proposed system. A change in the traditional GRU increases the learning efficiency of this model. The NexusOpti model with the E-GRU layer demonstrates a significant improvement in NDCG metrics. From Table 6, when comparing the NDCG@5 scores between the NexusOpti model and the baseline models, the NexusOpti achieves an NDCG@5 score of 0.7499, compared to 0.6188 for the existing GRU model. As shown in Tables 7 and 8, the improved performance of the E-GRU improved the Hit Ratio by approximately 10% and NDCG by approximately 13%. The use of the E-GRU also increased the overall learning rate of the proposed model.

**Table 7:** Comparison of GRU and E-GRU using Hit Ratio.

	HR@1	HR@2	HR@3	HR@4	HR@5
Existing GRU	0.328	0.6572	0.6764	0.7047	0.7297
E-GRU	0.4170	0.7747	0.7982	0.8175	0.8392

**Table 8:** Comparison of GRU and E-GRU using NDCG.

	NDCG@1	NDCG@2	NDCG@3	NDCG@4	NDCG@5
Existing GRU	0.4507	0.5885	0.5932	0.6066	0.6188
E-GRU	0.6085	0.7297	0.7332	0.7396	0.7499

The NexusOpti framework handles cases requiring multiple medications by recommending a ranked list of drugs based on factors like medical history, demographics, disease state, and drug interactions. The model incorporates hierarchical data from ICD and ATC ontologies to evaluate drug combinations, ensuring safety and therapeutic relevance. Evaluation was done using metrics with Hit Ratio and NDCG, which assess the quality of ranked recommendations. A negative sampling strategy helps distinguish appropriate drug combinations, and the inclusion of Cross-Sectional (CS) and Longitudinal (LT) features enhances prediction accuracy by considering both short-term and long-term interactions.

### Security analysis

Adversarial attacks can cause DL models to misclassify or provide inaccurate output. These attacks are caused by tiny, well-crafted perturbations in input data. If DL models trained on sensitive data, such as medical data, can unintentionally release sensitive information or allow attackers to deduce sensitive information from the model outputs, they may constitute a privacy risk. By ensuring that private data are not revealed during model training or inference, strategies such as differential privacy, federated learning, and secure multiparty computing can help reduce privacy issues. To avoid security treatment, integrating DL with ontology facilitates judgments that are better understood and explained using ontologies, which offer an organised and semantically meaningful representation of domain knowledge. Integrating ontology with DL improves trust and transparency in model behaviour by simplifying the understanding of why a certain prediction was produced. It captures domain information in a manner that is both reusable and transferable.

## Conclusion and future work

Medicine prescriptions play a critical role in healthcare, impacting treatment effectiveness, patient outcomes, and overall healthcare expenditures. Several factors are considered when prescribing medications, including patient characteristics, medical history, symptoms, drug interactions, and possible adverse effects. According to a thorough experimental analysis, the proposed method E-GRU demonstrates superior performance compared to the existing GRU model, with a E-GRU shows a 13% improvement over the GRAM baseline model in terms of NDCG@5 state-of-the-art techniques. The proposed methodology was implemented in Python, and a noticeable improvement in the evaluation was confirmed. This procedure involves constructing an advanced framework that uses DL techniques to create prediction models for prescription drugs. It incorporates several elements, including pharmacological information, medical history, patient demographics, and embeddings for medical codes such as ICD and ATC. A thorough training pipeline that manages data loading, model initialisation, optimisation, and assessment was implemented using the code. When the E-GRU model is incorporated into a broader pipeline, it exhibits better prediction performance and efficiency in determining intricate temporal linkages between a patient’s medication routes and the EHR. Dropout regularisation, bidirectional processing, and appropriate weight initialisation are some of the strategies used by GRU to address common problems with recurrent neural networks, such as overfitting and vanishing gradients. This facilitates the pretraining and fine-tuning stages to maximise the accuracy of the model. To evaluate the model’s performance and ranking accuracy, evaluation metrics such as loss, hit ratio (HR@K), and NDCG@K were used. This model is noteworthy because of its flexibility. It facilitates experimentation and optimisation depending on the datasets and requirements by allowing the customisation of the model hyperparameters, optimiser selection, and initialisation techniques.

In the future, data augmentation techniques specifically designed for EHR data should be studied and implemented to improve this framework. By adding synthetic samples to the training dataset or using methods such as noise injection and time warping, the variety and representativeness of the data can be increased, which lowers overfitting and boosts the model’s capacity to generalise. Furthermore, the input representation of the model can be improved through feature engineering initiatives aimed at obtaining informative features from patient demographics, medication characteristics, and medical histories. This enables more precise and accurate prediction. Additionally, the framework can be expanded to accommodate model ensemble methods, which involve combining several GRU models with various initialisations or topologies to obtain a collective prediction. In addition to improving prediction stability and reducing the possibility of model bias, ensemble approaches can take advantage of the complementary capabilities of separate models.

## Limitations

Despite the promising results obtained from the NexusOpti model, there are several limitations that need to be acknowledged. The dataset used in this study was relatively small, comprising only 3,000 patients. While the dataset is representative of certain clinical conditions, its limited size may affect the generalizability of the model. The model was trained for a relatively low number of epochs, specifically five epochs. While this was sufficient for initial experimentation, increasing the number of epochs may help further improve the model’s learning, especially when dealing with complex medical data. The performance evaluation of the NexusOpti model primarily relied on ranking metrics such as NDCG and Hit Ratio. While these metrics are commonly used in recommendation systems, they focus on the ranking quality and may not fully capture the clinical relevance or accuracy of the recommended medications.

## Data Availability

The data that support the findings of this study have been deposited in the repository Github.com. Raw data can be found following link. https://github.com/Scientistmkh/MarketScan.git.
